# Monopolar diathermy *versus* a vessel-sealing device for reducing postoperative drain output after simple mastectomy: randomized clinical trial

**DOI:** 10.1093/bjs/znae029

**Published:** 2024-03-18

**Authors:** Stephen Keelan, Gavin P Dowling, Trudi Roche, Aisling Hegarty, Matthew G Davey, Amenah A Dhannoon, Sorcha O’Grady, Eithne Downey, Jarlath Bolger, Michael Boland, Jan Sorensen, Colm Power, Abeeda Butt, Chwanrow Baban, Arnold D K Hill

**Affiliations:** Department of Surgery, RCSI University of Medicine and Health Sciences, Dublin, Ireland; Beaumont RCSI Cancer Centre, Beaumont Hospital, Dublin, Ireland; Department of Surgery, RCSI University of Medicine and Health Sciences, Dublin, Ireland; Beaumont RCSI Cancer Centre, Beaumont Hospital, Dublin, Ireland; Department of Surgery, RCSI University of Medicine and Health Sciences, Dublin, Ireland; Beaumont RCSI Cancer Centre, Beaumont Hospital, Dublin, Ireland; Department of Surgery, RCSI University of Medicine and Health Sciences, Dublin, Ireland; Beaumont RCSI Cancer Centre, Beaumont Hospital, Dublin, Ireland; Beaumont RCSI Cancer Centre, Beaumont Hospital, Dublin, Ireland; Beaumont RCSI Cancer Centre, Beaumont Hospital, Dublin, Ireland; Department of Surgery, RCSI University of Medicine and Health Sciences, Dublin, Ireland; Beaumont RCSI Cancer Centre, Beaumont Hospital, Dublin, Ireland; Beaumont RCSI Cancer Centre, Beaumont Hospital, Dublin, Ireland; Beaumont RCSI Cancer Centre, Beaumont Hospital, Dublin, Ireland; Beaumont RCSI Cancer Centre, Beaumont Hospital, Dublin, Ireland; Health Outcomes Research Centre, School of Population Health, RCSI University of Medicine and Health Sciences, Dublin, Ireland; Beaumont RCSI Cancer Centre, Beaumont Hospital, Dublin, Ireland; Beaumont RCSI Cancer Centre, Beaumont Hospital, Dublin, Ireland; Beaumont RCSI Cancer Centre, Beaumont Hospital, Dublin, Ireland; Department of Surgery, RCSI University of Medicine and Health Sciences, Dublin, Ireland; Beaumont RCSI Cancer Centre, Beaumont Hospital, Dublin, Ireland

## Abstract

**Background:**

Electrosurgical devices are commonly used during mastectomy for simultaneous dissection and haemostasis, and can provide potential benefits regarding vessel and lymphatic ligation. The aim of this prospective RCT was to assess whether using a vessel-sealing device (LigaSure™) improves perioperative outcomes compared with monopolar diathermy when performing simple mastectomy.

**Methods:**

Patients were recruited prospectively and randomized in a 1 : 1 manner to undergo simple mastectomy using either LigaSure™ or conventional monopolar diathermy at a single centre. The primary outcome was the number of days the drain remained *in situ* after surgery. Secondary outcomes of interest included operating time and complications.

**Results:**

A total of 86 patients were recruited (42 were randomized to the monopolar diathermy group and 44 were randomized to the LigaSure™ group). There was no significant difference in the mean number of days the drain remained *in situ* between the monopolar diathermy group and the LigaSure™ group (7.75 days *versus* 8.23 days; *P* = 0.613) and there was no significant difference in the mean total drain output between the monopolar diathermy group and the LigaSure™ group (523.50 ml *versus* 572.80 ml; *P* = 0.694). In addition, there was no significant difference in the mean operating time between the groups, for simple mastectomy alone (88.25 min for the monopolar diathermy group *versus* 107.20 min for the LigaSure™ group; *P* = 0.078) and simple mastectomy with sentinel lymph node biopsy (107.20 min for the monopolar diathermy group *versus* 114.40 min for the LigaSure™ group; *P* = 0.440).

**Conclusion:**

In this double-blinded single-centre RCT, there was no difference in the total drain output or the number of days the drain remained *in situ* between the monopolar diathermy group and the LigaSure™ group.

**Registration number:**

EudraCT 2018-003191-13 BEAUMONT HOSPITAL REC 18/66.

## Teaser

In this double-blinded single-centre RCT, there was no difference in the total drain output or the number of days the drain remained *in situ* between the monopolar diathermy group and the LigaSure™ group. Monopolar diathermy is at least as effective and significantly cheaper compared with LigaSure™.

## Introduction

Breast cancer is the most common cancer in women worldwide and surgery remains a crucial component of multimodal management^1^. While there has been a shift in contemporary surgical practice towards breast-conserving surgery, sometimes this is not clinically feasible^2^. In such patients, simple mastectomy is often performed. Simple mastectomy has several associated complications, with seroma formation being the most common^3,4^.

A seroma is a fluid collection that can accumulate in the dead space created when breast tissue is removed. This is partly due to lymphatic vessels being cut during surgery, resulting in lymphatic fluid leakage and subsequent accumulation^5,6^. The current rate of seroma formation is estimated to be 15.0%^4^. Seroma formation can lead to a prolonged hospital stay, discomfort, and impaired wound healing, and can be a source of delay for adjuvant therapy, particularly radiotherapy^7–10^. To prevent or minimize the risk of seroma formation, various techniques can be employed, including placing drains in the surgical bed, quilting sutures to close the dead space, and applying compression dressings. Whilst closed-suction drain placement is currently routinely used, with the aim of reducing seroma formation, there is a paucity of evidence to support this^11^. Conversely, prolonged drain placement can negatively affect a patient’s quality of life and risks introducing infection^12^. The choice of electrosurgical device has been shown to influence and potentially reduce seroma formation in other surgical procedures^13^. An electrothermal bipolar vessel-sealing system has been shown in some studies to decrease blood loss and drainage volume compared with electrocautery^13–17^. Other common complications after simple mastectomy include haematoma formation and surgical-site infection (rates of 0.6–7% and 5–6% respectively)^4^. Complications can also impact the length of time that postoperative drains remain *in situ*, reoperation rates, and postoperative pain, as well as contribute to delays in patients receiving adjuvant treatment.

Whereas the standard reference technique for simple mastectomy involves the use of monopolar electrocautery, newer electrosurgical devices, such as LigaSure™ and the harmonic scalpel, are commonly used during simple mastectomy for simultaneous dissection and haemostasis. Their potential benefits regarding vessel and lymphatic sealing are not known^18,19^. LigaSure™ (Medtronic, Dublin, Ireland) is an electrothermal bipolar vessel-sealing system that achieves haemostasis using a combination of pressure and electrothermal energy by denaturing collagen and elastin within the vessel wall and surrounding connective tissue. This haemostatic device ensures vessel sealing, with minimal thermal spread and limited tissue charring^19–21^.

The aim of this prospective RCT was to assess whether using a vessel-sealing device (LigaSure™) improves perioperative outcomes compared with monopolar diathermy when performing simple mastectomy.

## Methods

### Study design and participants

The CONSORT guidelines for randomized studies were utilized for this prospective single-centre RCT^22^. See the *Supplementary material* for the CONSORT checklist. This RCT was a standard two-group parallel-designed trial conducted at a high-volume surgical centre (Beaumont Hospital) in Dublin in the Republic of Ireland. Patients were randomized in a 1 : 1 ratio. All potential participants received an information leaflet and explicit informed written consent was obtained before enrolment. Patient demographics and biometrics, including sex, age, and BMI, were recorded at the time of enrolment. Ethical approval was obtained from the Beaumont Hospital Research Ethics Committee (REC: 18/66). This RCT opened for recruitment on 15 August 2019. The study completed recruitment on 11 November 2022 and the follow-up interval was completed on 23 November 2022.

### Inclusion and exclusion criteria

Patients aged 18 years or over, presenting for simple mastectomy, with or without a sentinel lymph node biopsy (SLNB), were considered for inclusion. All patients had to be classified as ASA grade I or II to warrant inclusion^23^. Exclusion criteria included axillary lymph node dissection, immediate breast reconstruction, and pregnancy/lactation.

### Randomization

After the consent process, randomization occurred using a digital randomization tool. A custom design to stratify for BMI greater than and equal or less than 25 kg/m^2^ was used, while maintaining a 1 : 1 randomization. This was developed to ensure equality in both groups. The patient and the nurse collecting postoperative drain outputs were blinded to the assigned study arm.

The operating surgeon was informed of the electrosurgical device to which the patient was assigned before surgery and proceeded according to specific and predefined guidelines.

### Simple mastectomy techniques

The procedure for simple mastectomy as per the study protocol involved the following steps. The surgeon makes an elliptical incision encompassing the nipple-areolar complex that can vary based on the size and location of the tumour. The surgeon then dissects the subcutaneous tissue to expose the breast tissue using the assigned diathermy device in the standard mastectomy plane down to the chest wall. The breast tissue is then dissected free from the pectoral muscles and chest wall. The specimen is then removed en bloc and sent as a histopathological specimen with standard suture markings of Long Lateral (LL), Short Superior (SS), and Double Deep (DD). The surgeon inserts a 7 mm Jackson–Pratt drain into the breast cavity to remove any excess fluid that can accumulate after surgery. The drain is typically placed through a separate incision in the anterior chest wall. Finally, the drain is secured to the skin using a silk 2-0 suture. Closure is completed in the usual manner using absorbable sutures, Steri-Strips™, and waterproof adhesive dressing.

Both groups received standard postoperative care that involved patients being educated in drain management and discharged with the drain *in situ*. Drains were removed in the outpatient department by a nurse when draining less than 30 ml per 24 h. Patients were educated before discharge on documenting the total drain volume in a daily drainage diary. Patient follow-up was in the outpatient department on postoperative day 5 and then weekly until drain removal.

### Outcomes

The primary outcome was the number of days the drain remained *in situ* after surgery. Secondary outcomes included operating time and complications, including seroma formation, haematoma formation, and wound infection.

### Power calculation

Sample size calculation was based on the primary outcome measure, that is the mean number of days the drain remained *in situ*. A 3-day difference in duration of drain *in situ* was considered clinically meaningful. The sample size calculation was based on the assumption that patients operated on with LigaSure™ would have a shorter duration of drain *in situ*. A pragmatic sample size was determined to test the hypothesis. Using Stata’s power calculation command, assuming a significance level of α = 0.05 and a power of 80%, a sample size of 86 participants was required (power two mean(s.d.) 14 11(5.5), one-sided). This sample size also supported analysis of the secondary outcomes that included operating time and complications.

### Statistical analyses

Continuous data are summarized as mean(s.d.) and categorical data as *n* (%). Analyses were performed on an intention-to-treat principle, retaining all participants in their randomized groups. Clinical and demographic information was correlated with the electrosurgical device used, using chi-squared and two-tailed *t* tests, as appropriate. The Shapiro–Wilk test was used to assess the distribution of data. All significance tests were two-tailed, with *P* < 0.05 indicating statistical significance. Data were analysed using SPSS^®^ (IBM, Armonk, New York, USA; version 26.0).

## Results

**Fig. 1:**
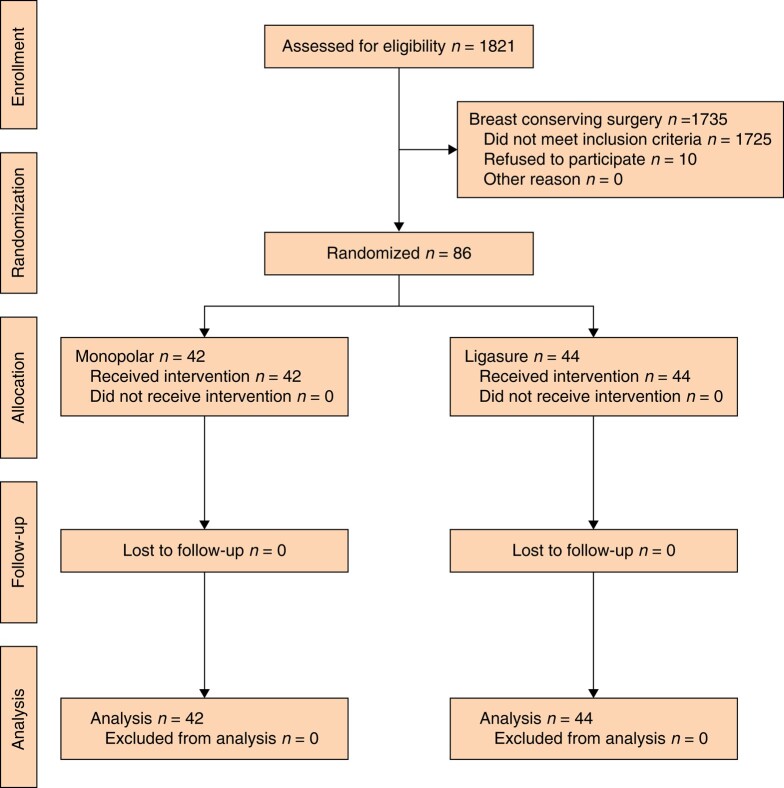
CONSORT diagram of the progress through the phases of this RCT

A total of 86 patients were recruited, all of whom were followed up and analysed. Details of enrolment, allocation, follow-up, and analysis are summarized in the CONSORT diagram (*Fig. 1*). After randomization, 42 patients were assigned to the monopolar diathermy group and 44 to the LigaSure™ group. Patients in both groups had similar demographics (mean age, mastectomy indication, number of patients undergoing SLNB, mean BMI, and mean breast weight), as summarized in *Table 1*.

**Table 1:** Patient demographics

	All (*n* = 86)	Monopolar diathermy (*n* = 42)	LigaSure™ (*n* = 44)
Age (years), mean (sd)	60.79 (1.6)	62.4 (13.3)	58.8 (16.7)
**Mastectomy indication**			
Completion	19 (22.1)	11 (26.1)	8 (18.2)
Primary	64 (74.4)	30 (71.4)	34 (77.3)
Prophylactic	3 (3.5)	1 (2.4)	2 (4.5)
SLNB	65 (75.6)	33 (78.6)	32 (72.7)
No SLNB	21 (24.4)	9 (21.4)	12 (27.3)
BMI (kg/m^2^), mean (sd)	27.89 (5.22)	27.61 (5.31)	28.15 (5.19)
Breast weight (g), mean (sd)	877.3 (515.30)	902.5 (575.09)	850.5 (460.5)

Values are *n* (%) unless otherwise indicated. SLNB, sentinel lymph node biopsy.

### Perioperative outcomes

**Fig. 2:**
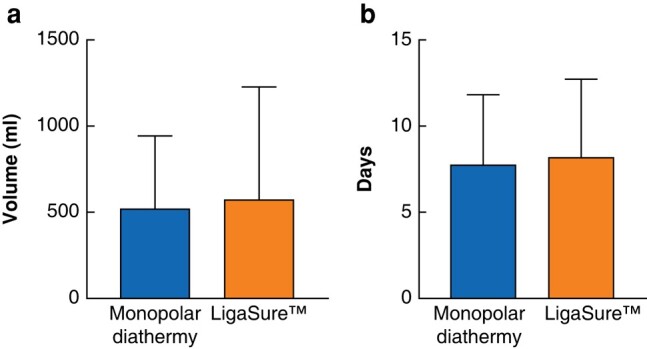
Drain output and number of days drain *in situ* **a** Mean total drain output. Monopolar diathermy = 523.5 ml and LigaSure™ = 572.8 ml; *P* = 0.694. **b** Mean number of days to drain removal. Monopolar diathermy = 7.75 days and LigaSure™ = 8.23 days; *P* = 0.613.

There was no significant difference in mean total drain output between the monopolar diathermy group and the LigaSure™ group (523.5 ml *versus* 572.8 ml respectively; *P* = 0.694) (*Fig. 2a*). There was also no significant difference in the mean number of days the drain remained *in situ* between the monopolar diathermy group and the LigaSure™ group (7.75 days *versus* 8.23 days respectively; *P* = 0.61) (*Fig. 2b*).

### Operating time

There was no significant difference in the mean operating time between the groups, for simple mastectomy alone (88.25 min for the monopolar diathermy group *versus* 107.5 min for the LigaSure™ group; *P* = 0.078) and simple mastectomy with SLNB (107.2 min for the monopolar diathermy group *versus* 114.4 min for the LigaSure™ group; *P* = 0.448). See *Table 2*. Notably, the time taken to carry out a simple mastectomy was a mean of 19 min longer using LigaSure™.

**Table 2:** Secondary outcomes in Monopolar versus LigaSure

	All (*n* = 86)	Monopolar diathermy (*n* = 42)	LigaSure™ (*n* = 44)	*P*
**Operating time (min), mean**				
Mastectomy only	–	88.25	107.5	0.078
Mastectomy + SLNB	–	107.2	114.4	0.448
**Complication**				
Seroma formation	4 (4.7)	2 (4.8)	2 (4.5)	0.944
Haematoma formation	2 (2.3)	2 (4.8)	0 (0)	0.139
Wound infection	5 (5.8)	2 (4.8)	3 (6.8)	0.683

Values are *n* (%) unless otherwise indicated. SLNB, sentinel lymph node biopsy.

### Complications

There was no significant difference in complication rates between the monopolar diathermy group and the LigaSure™ group. See *Table 2*. Both the monopolar diathermy group and the LigaSure™ group had a similar rate of seroma formation (4.8% and 4.5% respectively; *P* = 0.944). While not statistically significant, the rate of haematoma formation was higher in the monopolar diathermy group compared with the LigaSure™ group (4.8% *versus* 0% respectively; *P* = 0.139). The rate of wound infection requiring treatment with antibiotics was also not significantly different between the monopolar diathermy group and the LigaSure™ group (4.8% *versus* 6.8% respectively; *P* = 0.683).

## Discussion

Despite rapid advances in surgical haemostasis technology, the current surgical paradigm fails to provide a standardized approach for tissue cutting and sealing devices in breast cancer. Several factors currently influence a surgeon’s choice of diathermy equipment, such as ergonomics, cost, and outcomes. While several non-randomized studies have investigated the optimal sealing device for axillary surgery in breast cancer, there is a paucity of information available breast surgeons regarding which device makes a meaningful difference to patient outcomes for simple mastectomy. In one RCT by Park *et al*.^24^, LigaSure™ was found to significantly improve drainage volume and duration in comparison with monopolar diathermy. Park *et al*.^24^ showed favourable outcomes for the LigaSure™ group; however, owing to the diversity of procedures performed by Park *et al*.^24^, such as the inclusion of immediate reconstruction and/or axillary dissection, it was still unknown whether LigaSure™ improves outcomes for patients undergoing simple mastectomy alone.

In terms of the primary outcome of interest, there was no difference in the number of days the dain remained *in situ* between monopolar diathermy and LigaSure™ in the present trial. In keeping with this finding, *the total drain output* was similar in both study arms. Similarly, there were no statistically significant differences in the secondary outcomes between the monopolar diathermy group and the LigaSure™ group (that is rates of seroma formation, haematoma formation, and wound infection). The 2.3% and 5.8% overall rates of postoperative haematoma formation and wound infection respectively are in keeping with current international guidelines and demonstrate acceptability of either practice.

Another essential consideration is operating time and this has implications for both the patient and the surgeon^25^. Monopolar diathermy traditionally represents a relatively quick and easy method of achieving haemostasis^26^. Conversely, LigaSure™, with its smaller surface area and the need for opening and closing the device with each cut, logically represents a slower device. Although no statistically significant increase in operating time was observed in the present study, performing a mastectomy using LigaSure™ was a mean of almost 20 min slower than performing a mastectomy using monopolar diathermy (mean of 107.5 min *versus* 88.25 min respectively; *P* = 0.41). Maximum efficiency without compromising patient outcomes is the goal of most surgeons. The findings from the present study place monopolar diathermy ahead of the more modern cutting and sealing LigaSure™ device, with no compromise with regard to seroma formation.

In addition, monopolar diathermy saves over €300 in comparison with LigaSure™ (€5 *versus* €340 respectively); this, coupled with its ease of use, low complication rates, and faster operating time, make monopolar diathermy an attractive option. These are essential considerations for surgeons, as cost savings and health economics are increasingly important in modern healthcare resource management.

There are some limitations, which must be taken into consideration when interpreting the results. Whereas the patient is blinded to the arm to which they are allocated, the operating surgeon is aware of the participant’s arm, making them subject to unintentional bias. A degree of information bias must be considered present within the present study, as drain outputs were self-reported by participants. Similarly, the decision to drain a seroma is made by an individual consultant based on their clinical acumen, which may introduce a degree of observer bias. Notwithstanding these limitations, the single-centre double-blinded methodology increases the robustness of the results of the present trial, thus supporting the routine use of monopolar diathermy over the vessel-sealing device LigaSure™ when performing simple mastectomy.

Simple mastectomy continues to be a commonly performed procedure for invasive and *in situ* breast cancer, as well as risk-reducing surgery. Methods to improve postoperative complication rates are important for this patient cohort and evidence-based surgery should be adhered to when introducing new medical devices. This RCT shows that, whilst both monopolar diathermy and the vessel-sealing device LigaSure™ are safe and effective for carrying out simple mastectomy, there are no significant differences in rates of seroma formation, haematoma formation, and wound infection between these devices. Monopolar diathermy represents a safe, cost-effective, and well-established sealing method that should remain as the first-line choice for surgeons when performing simple mastectomy.

## Supplementary Material

znae029_Supplementary_Data

## Data Availability

Data will remain within the Department of Surgery, RCSI for an interval of 7 years after study closure. Data available for audit/interrogation. Participants were asked to consent to future research within this field of research. Data can be made available for future research in this capacity.
